# Toward an Emotioncy Based Education: A Systematic Review of the Literature

**DOI:** 10.3389/fpsyg.2021.727186

**Published:** 2021-08-05

**Authors:** Mir Abdullah Miri, Reza Pishghadam

**Affiliations:** English Department, Ferdowsi University of Mashhad, Mashhad, Iran

**Keywords:** emotioncy, avolvement, exvolvement, involvement, senses, emotions, emotioncy-based education

## Abstract

The current systematic review summarizes the growing body of literature on the concept of emotioncy. It presents a synthesis of 61 studies discussing emotioncy related topics. The aims were to examine, interpret, and synthesize results about emotioncy to generate an in-depth and holistic discussion of the key routes of emotioncy based education and the different influencing factors at policy and practice levels. The review revealed that emotioncy has been explored in different disciplines, particularly English language teaching, Persian language teaching, neuroscience, and psychosociology. It was shown that although both empirical and theoretical studies have been conducted on emotioncy, there is abundant room for future studies to use various research methodologies and scopes. The review offers a few data-driven pedagogical implications on emotioncy-based education. The authors argue that emotioncy warrants closer scrutiny in different disciplines.

## Introduction

Emotioncy (emotion + frequency) is defined as sense-induced emotions which can relativize cognition (Pishghadam et al., 2013). Research on emotioncy highlights the importance of senses in relativizing cognition (Pishghadam et al., 2013; Pishghadam, 2015). It is paramount to emphasize the role of senses when discussing emotioncy because senses connect people with the outside world. A combination of senses (multisensory or sense combinations) offers opportunities for inclusive learning (Katai, 2011; Shayesteh et al., 2019). According to Pishghadam et al. (2013), the frequency of sensory experience awakens and moves emotioncy to evoke emotions through the senses, which can relativize cognition. Pishghadam et al. (2013) also postulated that people's emotional levels, mainly caused by their senses, are different. They also asserted that the number of senses involved in a person's task involvement influences the level of emotioncy. For example, in vocabulary acquisition, the type and number of senses involved in the task (i.e., hearing, seeing, etc.) impact vocabulary learning and retention.

The emotioncy notion presents that “individuals can construct their idiosyncratic understanding of the world through their senses” (Pishghadam et al., 2016, p. 14). Suppose that some L2 learners are not familiar with the word pitaya. Because they have never heard, seen, touched, or tasted the fruit, their emotioncy level would be zero (null). On the other hand, L2 learners with more experience of the word in terms of senses involved would have a higher emotioncy level.

Furthermore, the emotions and the sensory inputs people receive from the environment influence their understanding of reality and perception of the future (Pishghadam et al., 2016), as such perceptions stem from the sensory data they experience in life (Dewey, 1906). How a person connects to self, individuals, and other creatures is mainly because of the existing senses involved in their experiences (Pishghadam and Shakeebaee, 2020). Therefore, it can be argued that individuals are continuously evolved by different senses, emotions, and feelings they experience.

**Figure 1:**
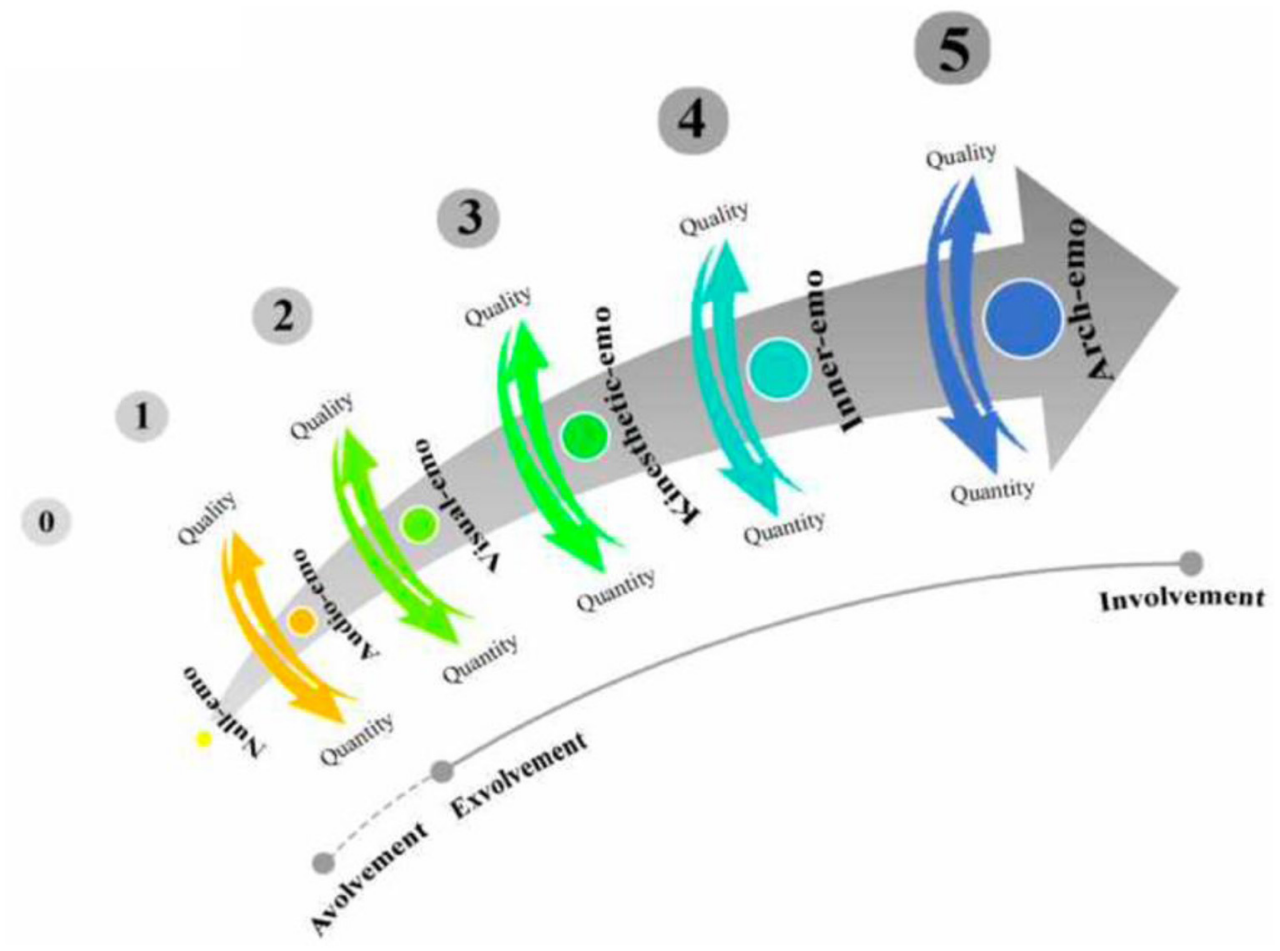
Emotioncy matrix. Adapted from Pishghadam (2015).

Devising the emotioncy concept into a six-level matric, Pishghadam (2015) illustrated a person's emotioncy level toward a particular phenomenon or notion (see Figure 1).

Moreover, Pishghadam (2015) categorized and named each emotioncy type with unique level(s)/kind(s). As shown in Table 1, the six devised emotioncy levels are categorized into three types: avolvement, exvolvement, and involvement. A description of each emotioncy level is presented in the following adapted table.

**Table 1:** Type and kinds of emotioncy.

**Type**	**Kind**	**Score**	**Experience**
Avolvement	Null emotioncy	0	When an individual has not heard about, seen, or experienced an object or a concept.
Exvolvement	Audio emotioncy	1	When an individual has merely heard about a word/concept.
	Visual emotioncy	2	When an individual has both heard about and seen the item.
	Kinesthetic emotioncy	3	When an individual has touched, worked, or played with the real object.
Involvement	Inner emotioncy	4	When an individual has directly experienced the word/concept.
	Arch emotioncy	5	When an individual has done research to get additional information.

*Adapted from “Conceptualizing Sensory Relativism in Light of Emotioncy: A Movement beyond Linguistic Relativism,” by Pishghadam et al. (2016). Copyright 2015 by IJSCL*.

In this model, emotioncy level starts with avolvement (null emotioncy), continues to exvolvement (audio emotioncy, visual emotioncy, kinesthetic emotioncy), and involvement (inner emotioncy and arch emotioncy), which includes the avolvement and exvolvement types. In this sense, each emotioncy level adds to its previous level. As written in Table 1, the emotioncy kind(s) in each emotioncy type encompasses the previous emotioncy kind. For example, when the individual becomes exvolved in something, it means that the person has passed the avolvement stage. Besides, an individual with visual emotioncy has audio emotioncy as well. Hence, “moving the latter of emotioncy can provide an individual with a more thorough emotional experience of the object or concept” (Khoshsaligheh et al., 2018, p. 78).

As the table demonstrates, each emotioncy level builds upon the previous level and includes the features of the preceding emotioncy kind. To illustrate, an involved person has surpassed the exvolved and avolved people because the experience “provide[s] an individual with a more thorough emotional experience of the object or concept” (Khoshsaligheh et al., 2018, p. 78).

**Figure 2:**
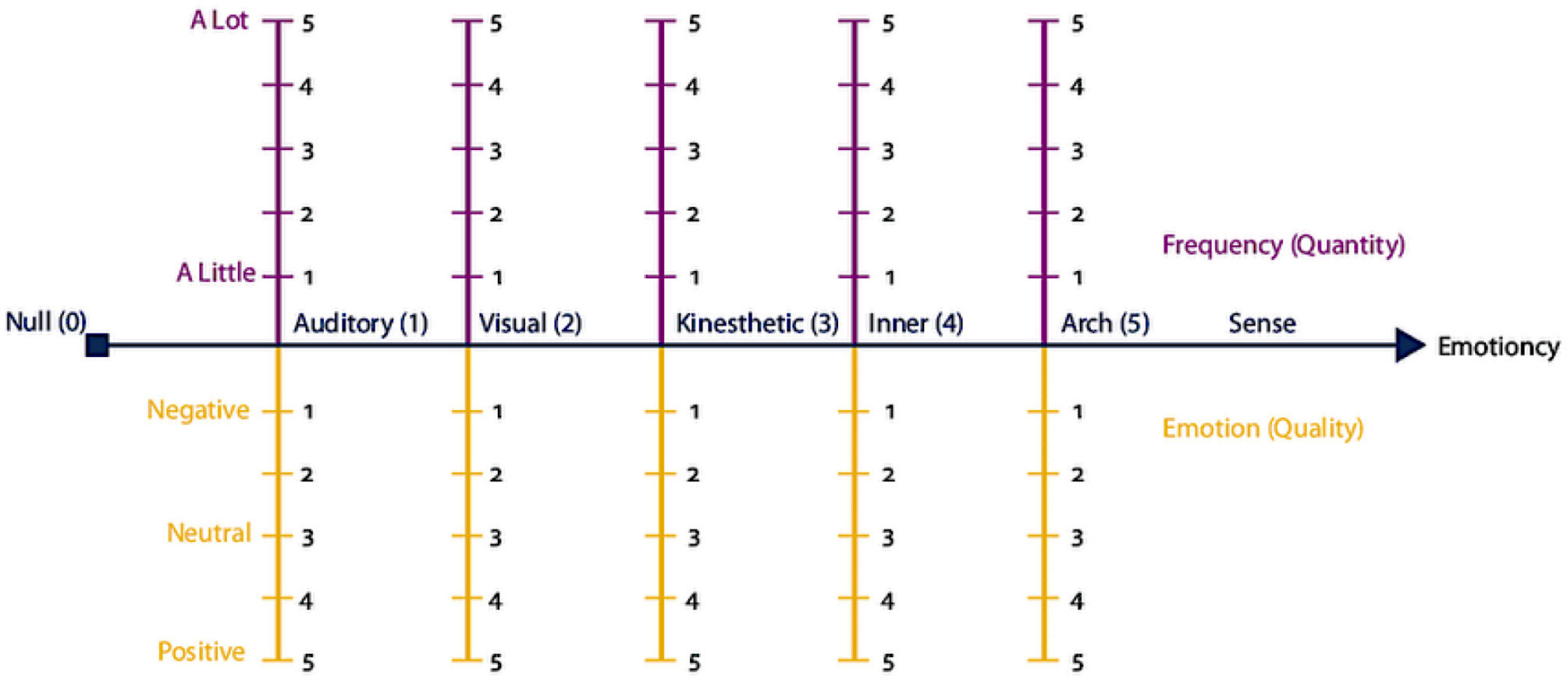
Emotioncy metric. Adapted from Pishghadam (2016a).

Later, Pishghadam (2016a) introduced an emotioncy metric for emotioncy level measurement. The metrics can measure frequency and emotion at the same time. The frequency scale indicates an individuals' exposure to a particular sense; it ranges from a little to a lot. On the contrary, the emotion valence of a person is measured through the emotion scale, with three levels: negative, natural, and positive. The former scale (frequency) is quantitative because it shows the amount of exposure to a specific sense, ranging from “a little” to “a lot,” while the latter (emotion) is qualitative as it indicates the emotion valence for the particular object, ranging from negative to positive. The emotioncy metric is further demonstrated in Figure 2.

Pishghadam et al. (2013) further expressed that in second language teaching, individuals need to go “beyond pure and conventional contextualization… and move toward emotionalization” (p. 11). They introduced emotionalization and inter-emotionality concepts as the key relevant concepts to emotioncy. To illustrate, they noted that in teaching vocabulary, the role of lexical emotions should be magnified. Considering the continuum of emotioncy matrix, when learners are involved in a task like learning new words, their retention rates increase because more senses are involved.

**Figure 3:**
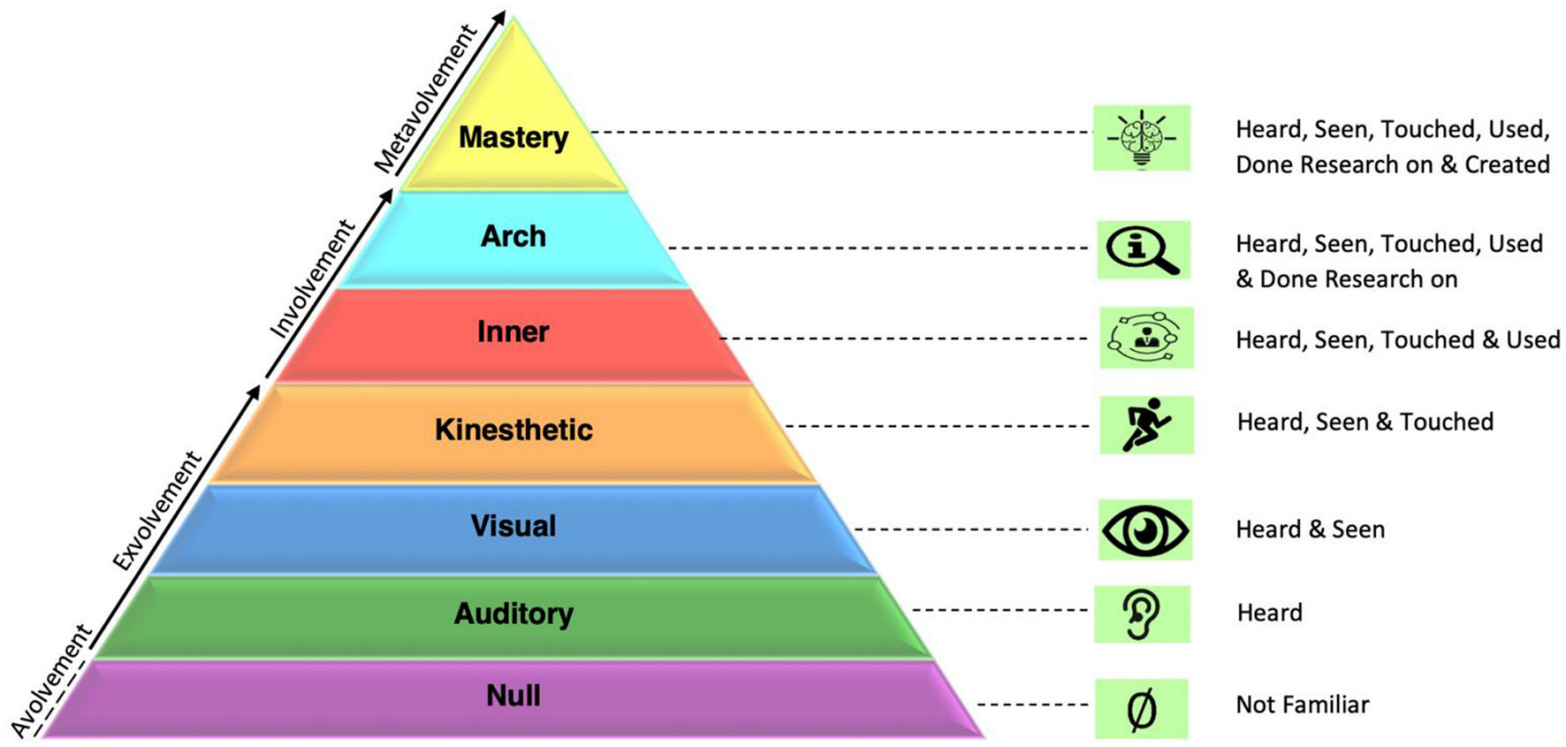
Emotioncy pyramid.

Later, the emotioncy matrix was extended by Pishghadam et al. (2019a). They added a new level, metavolvement, to the matrix. This is the level that shows an individual's mastery, being able to develop and produce content. This is considered the ultimate level of the emotioncy level because it feeds other levels (e.g., exvolvement, involvement). The metavolvement stage and its features are depicted in Figure 3.

Due to the various external and internal environmental factors, individuals may change their emotioncy level. This shift could be both forward and backward along with the emotioncy continuum levels. For example, someone involved in a task might get exvolved or metavolved with time (Pishghadam and Shayesteh, 2016). Hence, human socialization processes and internal and external mandates could influence their emotioncy levels in one way or another.

Research on the psychology of emotions (see Greenspan, 1992, Pishghadam et al., 2013) indicates that teachers are expected to carry out various emotional roles in the classroom. Hence, employing the concept of emotioncy, Pishghadam et al. (2019b) introduced the role of teachers as envolvers. Considering the different types of emotioncy from avolvement to metavolvement, these scholars postulated that “teachers seem to adopt an envolving role in which they avolve, exvolve or involve the learners in different classroom practices” (Pishghadam et al., 2019b, p. 41).

In educational psychology, teachers are encouraged to carry out some degree of emotions in their teaching practices (Pishghadam et al., 2013). Through this lens and emotioncy model, teachers are considered to have the role of envolvers in the classroom (Pishghadam et al., 2019a). Teachers can help learners to increase their emotioncy levels toward a concept.

## The Present Study

To date, the literature depicts no systematic review of existing research on emotioncy. Against this background, this article critically reviews existing literature (*n* = 61) on emotioncy from 2013 to April 2021. This review study has manifold purposes. Firstly, as the first attempt in current language education, educational psychology, and psychosociology research, the study seeks to examine, interpret, and synthesize results about emotioncy to generate an in-depth and holistic discussion of the key routes of emotioncy based education and the different influencing factors at policy and practice levels. Secondly, this review aims to synthesize the previous research findings to offer practical implications for teachers, teacher educators, and policymakers. Thirdly, comparing the reviewed studies on emotioncy based on their research methodologies and scopes, the paper aims to identify the gaps in emotioncy research and present future research directions.

Considering these research objectives, this study aims to answer four major questions: (1) What are the main themes of existing literature on emotioncy? (2) What disciplines have been involved in emotioncy research literature? (3) What research methodologies were employed by previous researchers on emotioncy? What are the future research directions on emotioncy?

## Research Methodology

### Literature Search and Selection

Rigorous systematic reviews entail some key features, including (1) clear and specific research question(s); (2) systematic research search; (3) clear study inclusion and exclusion criteria; (4) mythological quality assessment of studies; (5) bias reduction strategies in study selection and review process; (6) methodological transparency for the review process (Evans and Benefield, 2001). Guided by the research purpose and research questions, we followed these systematic review principles.

We collected the literature for this review from different digital databases (i.e., Google Scholar, ProQuest, ResearchGate, Academia, and Ferdowsi University of Mashhad Library) utilizing various keywords including emotioncy, deemotioncy, emotionalization, avolvement, exvolvement, involvement, and the Persian equivalent of these key terms because we reviewed the published Persian literature for this study as well. The inclusion and exclusion criteria for selecting the relevant studies were employed to identify studies on emotioncy from 2013 to April 2021. Both empirical and theoretical studies, including MA theses, Ph.D. dissertations and conference proceedings, published in English and Persian peer-reviewed journals were considered. Website reports and papers were not considered and included. Besides, of the 62 published studies on emotioncy, one study written in French was excluded from this study because the researchers did not know French.

### Analysis Procedures

**Figure 4:**
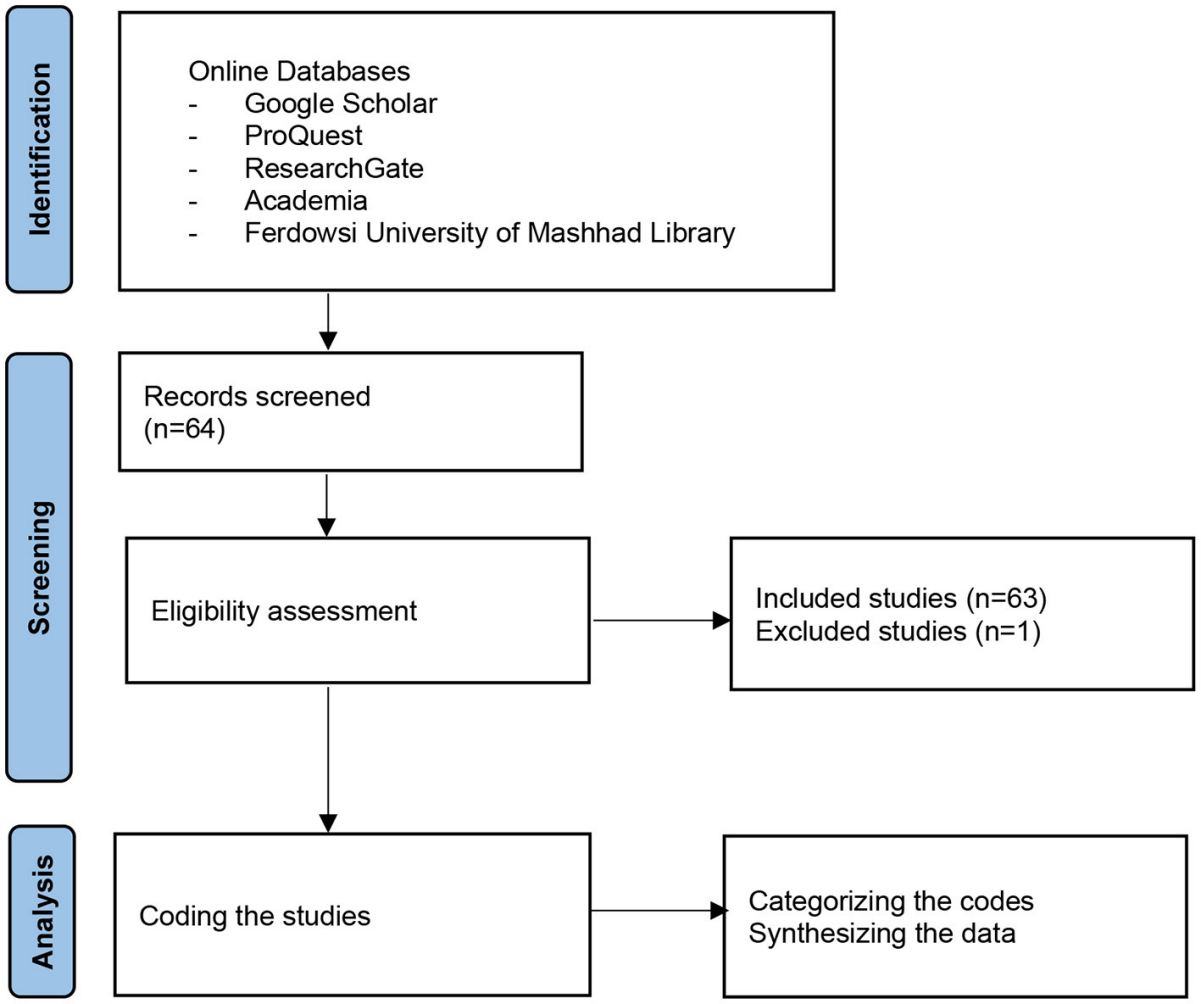
PRISMA flow chart.

We reviewed and analyzed the research studies by skimming them, searching for keywords, and focusing on how emotioncy was being studied. We created a table as a tool for keeping track of the related published works on emotioncy. The table was used as an analytical tool during the coding process, which included identifying the repeated concepts and ideas in the selected literature, resulting in finding the emergent themes. The initial codes were used to generate major categories for our findings (Merriam, 2009). Afterward, the studies were reexamined and synthesized against those categories. The Preferred Reporting Items for Systematic Reviews and Meta-Analysis (PRISMA) flow diagram (Figure 4) demonstrates the procedures.

## Findings

The reviewed studies (*n* = 61) demonstrated that the concept of emotioncy as a new strand has received a lot of attention in various disciplines. Researchers have conducted different types of theoretical and empirical studies on emotioncy. In the following paragraphs, the findings are presented based on the research questions.

### Discipline Related Emotioncy Studies

Among the 61 reviewed studies, 14 studies were theoretical, meaning that no data had been collected from participants, while the remaining studies (*n* = 47), including master's theses (*n* = 13) and Ph.D. dissertations (*n* = 6), were empirical. Most of the theoretical studies introduced new concepts and models. For example, cultuling analysis (Pishghadam et al., 2020a), sensory capital in education (Pishghadam et al., 2019c), extending the boundaries of multisensory teaching (Shayesteh et al., 2019), emotioncy tensions (Pishghadam, 2016b), EBLI (Pishghadam et al., 2013), emotioncy profile (Pishghadam et al., 2020b), and so forth. were introduced in the theoretical published research on emotioncy.

**Figure 5:**
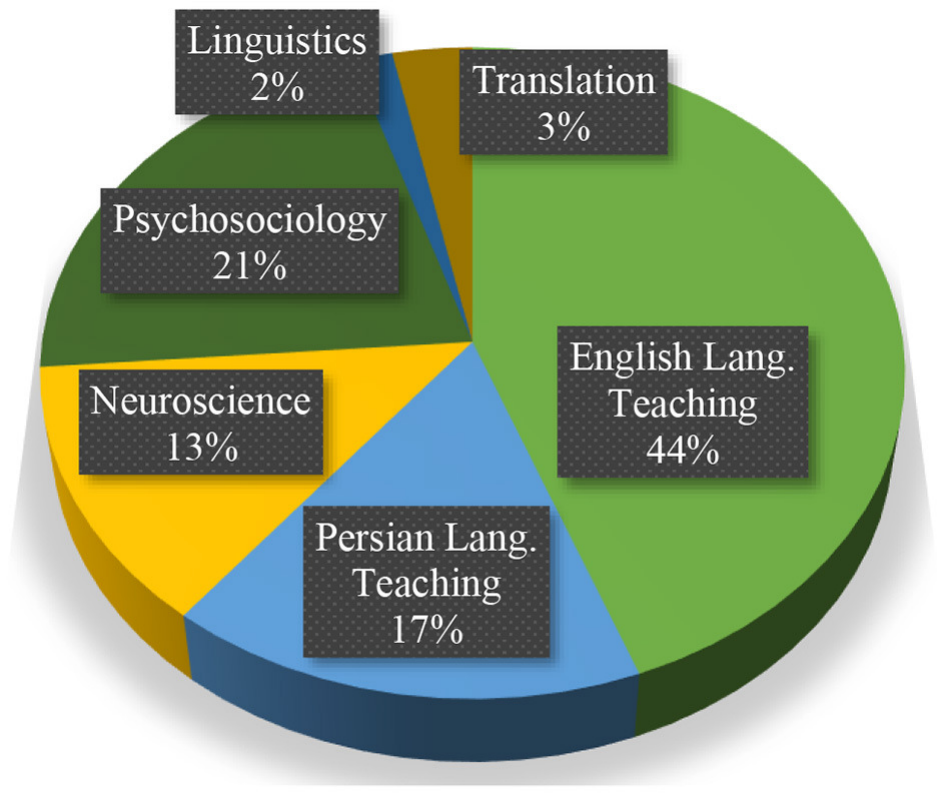
Emotioncy studies in different disciplines.

We categorized the reviewed studies into six major disciplines, namely English language teaching (*n* = 27), neuroscience (*n* = 8), Persian language teaching (*n* = 10), linguistics (*n* = 1), translation (*n* = 2), and psychosociology (*n* = 13). Worthy of note is that most of the studies could fall under multiple disciplines. For example, Jajarmi and Pishghadam (2019) study, entitled “The Effect of Word Repetition on Language Comprehension and Retention in Light of Emotioncy- Based Language Instruction (EBLI): An Event-Related Brain Potential (ERP) Study on Semantic Processing of a Sentence” could be listed under English language teaching discipline as well. However, since ERP was used to investigate the effect, we listed it under the neuroscience discipline. The same is true with many of our reviewed studies. Figure 5 presents the percentage of reviewed published literature on emotioncy based on the categorized disciplines.

### Employed Research Methodologies in Emotioncy Literature

**Figure 6:**
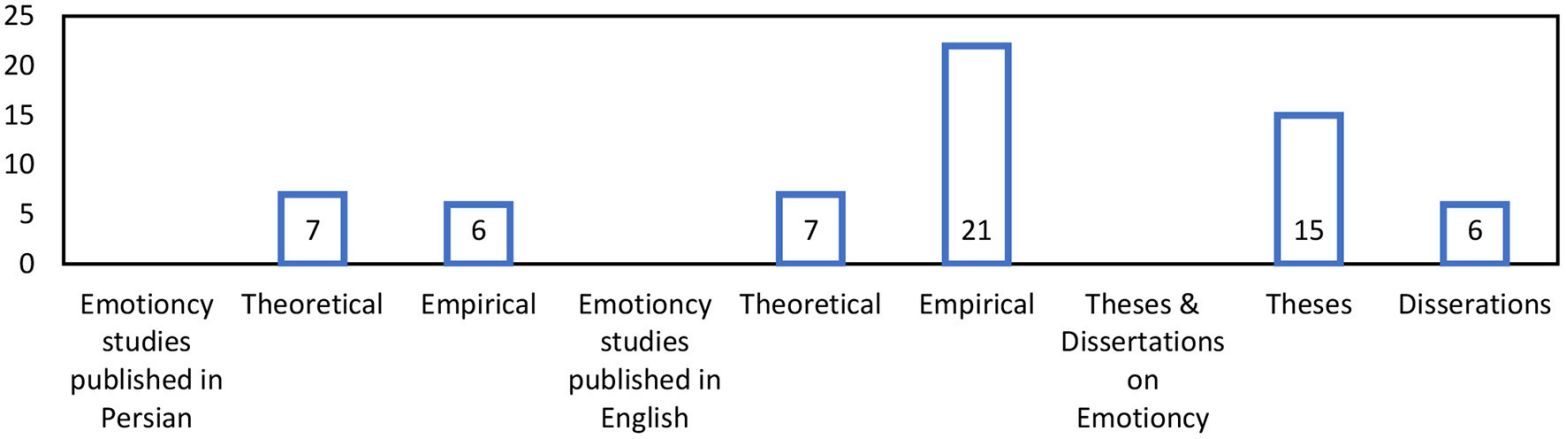
Published emotioncy research types.

Among the reviewed emotioncy studies (*n* = 61), there were 14 theoretical and 47 empirical studies. Figure 6 depicts the number of emotioncy master's theses (*n* = 13), Ph.D. dissertations (*n* = 6), published studies in English (*n* = 29) and published studies in Persian (*n* = 13).

The empirical studies were further categorized in terms of their research designs.

**Figure 7:**
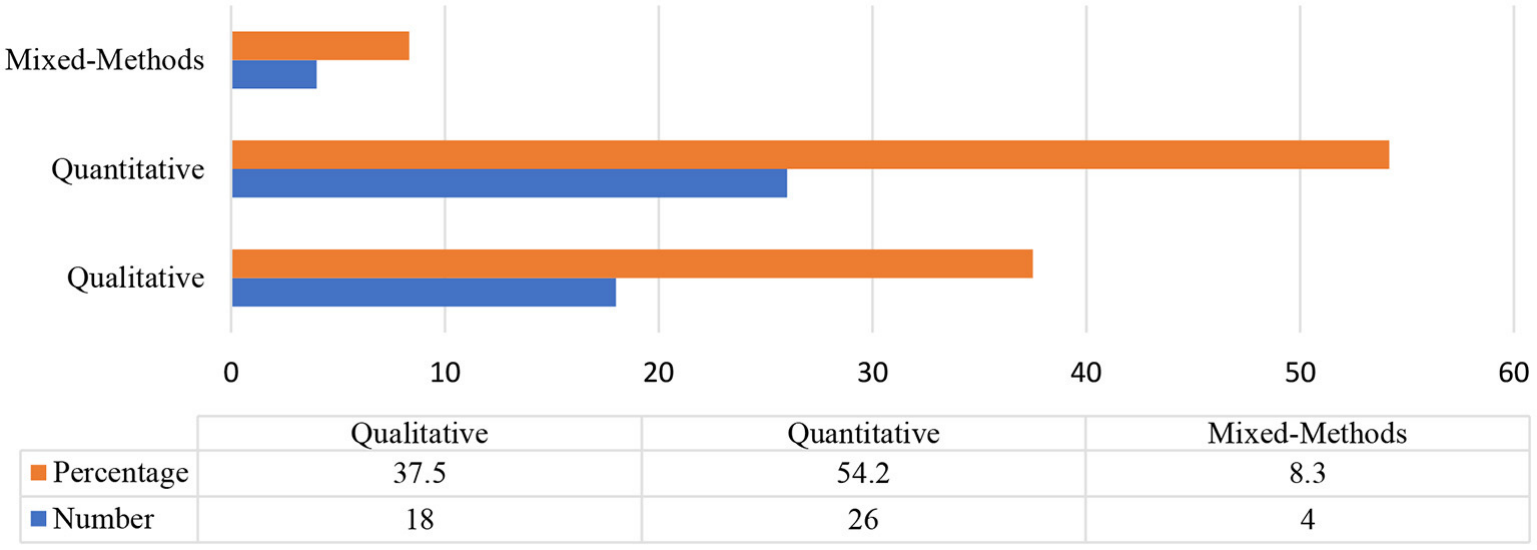
Research designs employed in empirical emotioncy studies.

As Figure 7 demonstrates, most of the conducted empirical studies on emotioncy were quantitative with 52.2%; only 8.3% of the studies were mixed-methods, which is to say both small-scale and large-scale studies were conducted. The data collection length among these studies ranged from 1 month to 2 years.

Our review of the studies indicated that various data collection methods, namely interview, scales (e.g., neophobia, handedness, emotioncy, Wechsler Adult Intelligence, self-identity changes, language learning orientation), document sources, and experimental data collection methods (e.g., event-related potential) were used. However, worthy of note is that no study on emotion has collected observation data.

In terms of data analysis approaches, most of the reviewed studies were quantitative (*n* = 26). The quantitative studies mostly used SPSS and AMOS for data analysis; many also employed structural equation modeling (SEM) to investigate the multivariate causal relationship between constructs. On the contrary, the qualitative studies mostly employed content analysis for analyzing their data.

### Major Themes of Existing Emotioncy Literature

Our analysis of the results illustrated that emotioncy research has received a lot of attention in various fields. Researchers have explored the concept of emotioncy from different lenses for various purposes in different disciplines. In the following table, we list major research topics and focus conducted on emotioncy in various disciplines.

Research on emotioncy has received considerable attention and added to the body of literature in different fields. As demonstrated in Table 2, a broad range of topics have been investigated in the six mentioned disciplines in light of emotioncy. The neuroscience related studies explored topics related to vocabulary retention (Jajarmi, 2019; Shayesteh, 2019; Seyednozadi, 2021) reading engagement, reading comprehension, and reading anxiety (Hamedi, 2019; Shayesteh et al., 2019), learners' emotions (Tabatabaee Farani, 2019), and linguistic congruency (Seyednozadi, 2021), and multisensory involvement (Pishghadam and Shayesteh, 2017). These research studies have added to the body of literature to a large extent, but the field is still in its infancy related to emotioncy; there is a lot to explore in this area, especially regarding the multidisciplinary focus of language education and neuroscience.

**Table 2:** Themes explored in emotioncy studies.

Neuroscience	– Language comprehension and retention – Reduce foreign language reading anxiety – Visual attention and lexical involvement – Responses to various degrees of sensory involvement	– Linguistic Congruency – Reading achievement/comprehension – Reading engagement – Learners' emotions – Neurocognitive foreign language comprehension – Word retention
English Language Teaching	– Emo-sensory competence – Teacher burnout – Community of practice – Life syllabus and emotionalization – Willingness to read – Willingness to communicate – Teacher as envolver – Emotion-based language instruction	– Test bias – Text readability – Vocabulary retention – Readability measurement – Contextualization – Emotionalization – Sensory relativism – Flow and reading comprehension
Persian Language Teaching	– Content analysis of Persian books – Sensory relativity – Flow and learning styles – Emotioncy-based teaching – Learners' emotions	– Teacher sensory awareness – Persian neologisms and loan words – Teaching Persian to non-Persian speakers – Lingua-cultural concept of nāz
Linguistics	Linguistic bias (i.e., judgment)	
Translation	Text translation accessibility	
Psychosociology	– Emotioncy profile – Individuals and social attitudes – Teacher-student communication model – Emo-sensory intelligence – Willingness to communicate – Cultural weight – Controlling causal decisions	– Sensory capital – Multisensory teaching – Life language model of emotioncy – Culture teaching strategies – Identity tensions – Cultuling (culture in language)

Additionally, the reviewed studies in English language teaching revealed that emotioncy has received more attention than the other five fields. The researched topics ranged from language skills (i.e., willingness to read, willingness to communicate, readability measurement, vocabulary retention) (see Pishghadam and Shayesteh, 2016; Shahian et al., 2017; Borsipour et al., 2019; Makiabadi, 2020) to life syllabus (Shakeebaee, 2020), emotionalization (Shakeebaee et al., 2017), teacher emotional labor and teacher burnout (Momenzadeh, 2020), test bias (Pishghadam et al., 2017a), emo-sensory capital (Pishghadam et al., 2018a) and Emotion-based language instruction (Pishghadam et al., 2013). There is abundant room for future studies on other areas of English language teaching, particularly other language skills, in light of emotioncy study.

In connection with Persian language teaching studies, topics related to sensory awareness and sensory relativity (Pishghadam and Ebrahimi, 2018), lexical words (Pishghadam et al., 2017b,c), content analysis (Jahani, 2020) and learner emotions (Ebrahimi et al., 2018) were explored. Similarly, studies related to psychosociology concentrated on emotioncy profile (Pishghadam and Abbasnejad, 2017a), cultural weight (Pishghadam and Firoozian Pour Esfahani, 2018), cultuling (Pishghadam et al., 2020a), causal decisions controlling (Pishghadam and Abbasnejad, 2017a), identity construction and social attitudes (Pishghadam, 2016b).

On the contrary, the concept of emotioncy in linguistics and translation warrants closer scrutiny since very few studies are conducted in these disciplines, focusing on linguistic bias (Pishghadam and Abbasnejad, 2017b) as well as text translation accessibility (Sadat Heiazian, 2020) and dubbing preference (Khoshsaligheh et al., 2018). We are likely to witness considerable progress in emotioncy research in the next decade as the topic merits further study.

## Discussion and Conclusion

Based on the review findings, it is clear that emotioncy has broadened its scope since 2013. Due to its multidisciplinary nature, the findings showed that emotioncy as a new research strand not only has incorporated a focus on language teaching but also has delved into various topics in neuroscience, linguistics, psychosociology, and translation.

In order to strike a balance between theory and practice, we discuss three major pedagogical implications suggested by the reviewed emotioncy published literature. Our contention is to offer macro-pedagogical teaching ideas related to emotioncy based education. Since the following educational concepts (EC) are not limited to one particular field, we think they could be useful for teachers and practitioners in different disciplines.

### Multisensory Teaching

One of the common pedagogical implications in the reviewed studies was related to multisensory teaching in facilitating learning. Nine studies recommended the creation of a sensory-rich environment (Shayesteh, 2019) by adding more sensory cues to the teaching topics (Seyednozadi, 2021) and engaging learners in more sensory inputs (Hamedi, 2019). In the absence of sensory experiences, long-term memory might not be enhanced easily (Tabatabaee Farani et al., 2019) as the measurement of sensory loads will be difficult (Akbari, 2020) and the presentation of concepts might mostly depend on verbal associations (Shayesteh, 2019). Hence, teachers are encouraged to incorporate multiple senses into their teaching practices to help learners increase their level of emotioncy.

### Learners as Envolvees

Drawing upon Pishghadam et al.'s (2019a) concept of teachers as envolvers, several studies suggested that teaching should be perceived as a transvolvement practice, creating, and cultivating a safe space for increasing students' emotioncy levels. One way to achieve that goal is to consider learner backgrounds in all stages of a lesson: preparation (Karami et al., 2019), teaching, and assessment (Shahian, 2020). Teachers need to be aware of their role as envolvers since they can significantly influence the senses and emotions of learners toward a particular concept. In the meantime, *learners as envolvees* need to understand that the emotional connections they make with the topics discussed in the class affect their learning. Hence, teachers need to consider the concepts their learners want to be envolved in when designing their course syllabi and lesson plans.

### Sources of Emotions in Teaching

In conjunction with Pishghadam's (2016a) concept of sensory motivation, some studies offered persuasive evidence for the role of sense-related emotions in learner motivation (Makiabadi, 2020) and student achievement (Jajarmi, 2019). The studies suggested that students' level of emotioncy and classroom participation highly depend on the teachers' application of senses and emotions (Pishghadam et al., 2013; Gholami, 2020). Teachers are expected to enrich their teaching by paying attention to senses and emotions learners might experience due to several sources of emotions, such as “teacher emotions, student emotions, emotional climate, task-induced emotions, environment-induced emotions, language-induced emotions, and sense-induced emotions” (Pishghadam, 2021, p. 19). Identifying the sources of emotions in teaching would enable learners to be immersed in the learning process (Pishghadam et al., 2017d). Worthy of note is that learners need to know the logic behind the emosensory, multisensory, and involvement-related tasks.

The analysis highlighted that emotioncy has received little attention in some disciplines (e.g., linguistics, translation). Future researchers could consider using the emotioncy model to explore topics not only in these two disciplines but also in the education field. Additionally, our review reports that multiple data sources, particularly observations, should be conducted to expand the research area related to emotioncy. Special attention can be paid to multidisciplinary and cross-cultural studies because the reviewed studies were done mainly by Iranian researchers in the Iranian context. Although there were a few studies collecting data from non-Persian speakers, further research on the notion of emotioncy in various contexts is greatly needed.

There is a need to incorporate emotioncy model into policy and planning. Future researchers could explore the extent the implemented policies and plans are aligned with emotioncy model. Currently, the Conceptual Model of Education used at Ferdowsi University of Mashhad, Iran, as an example, encompasses some of the components of emotioncy model. A critical investigation of the alignment between policy and practice in light of emotioncy model could add to the body of literature on this concept.

The most important limitation in the current study lies in the fact that the review was restricted to only investigating existing research studies on emotioncy in Persian and English languages, which might have influenced the findings. Since the emotioncy construct is novel, living in its own infancy, it needs to be examined from different perspectives. In fact, the emotioncy model focuses on one combination and order of senses, leaving other variations of senses untouched. Moreover, based on the model, one does not know the emotional valance of the concepts, which might be an important factor in experiencing and knowing the world around us.

## Data Availability Statement

The original contributions presented in the study are included in the article/supplementary material, further inquiries can be directed to the corresponding authors.

## Author Contributions

MM and RP developed and finalized the research design of the study. Under the supervision of RP, MM collected and analyzed the data. Next, MM wrote the first draft of the manuscript. RP reviewed the manuscript and provided feedback and also contributed to the manuscript revision, proofreading, and editing. Both authors contributed to the article and approved the submitted version.

## Conflict of Interest

The authors declare that the research was conducted in the absence of any commercial or financial relationships that could be construed as a potential conflict of interest.

## Publisher's Note

All claims expressed in this article are solely those of the authors and do not necessarily represent those of their affiliated organizations, or those of the publisher, the editors and the reviewers. Any product that may be evaluated in this article, or claim that may be made by its manufacturer, is not guaranteed or endorsed by the publisher.
